# Supplementary material for: Nodeless Superconductivity in Kagome Metal CsV3Sb5 with and without Time Reversal Symmetry Breaking
Source: Nano Lett. 2023 Jan 20;23(3):872–9. doi: 10.1021/acs.nanolett.2c04103 (PMC9912374; doi:10.1021/acs.nanolett.2c04103)
Supplement: Supplementary file 1 — nl2c04103_si_001.pdf [file nl2c04103_si_001.pdf]

Supporting Information for "Nodeless superconductivity in kagome metal CsV_3Sb_5 with and without time reversal symmetry breaking"

Wei Zhang,[1] Xinyou Liu,[1] Lingfei Wang,[1] Chun Wai Tsang,[1] Zheyu Wang,[1] Siu Tung Lam,[1] Wenyan Wang,[1] Jianyu Xie,[1] Xuefeng Zhou,[2] Yusheng Zhao,[2] Shanmin Wang,[2] Jeff Tallon,[3] Kwing To Lai*,[1,4] and Swee K. Goh†[1]

[1] *Department of Physics, The Chinese University of Hong Kong, Shatin, Hong Kong, China*
[2] *Department of Physics, Southern University of Science and Technology, Shenzhen, Guangdong, China*
[3] *Robinson Institute, Victoria University of Wellington, P.O. Box 600, Wellington, New Zealand*
[4] *Shenzhen Research Institute, The Chinese University of Hong Kong, Shatin, Hong Kong, China*

Supporting Information S1:

Temperature dependence of resistivity for selected bulk and thin flake samples

We present additional $\rho(T)$ data to support the construction of Figs. 1c and 2c in the main text. Figure S1 displays four additional datasets for the bulk sample while Fig. S2 shows four extra datasets for the thin flake. We choose the datasets carefully to cover the widest range of RRR values. The residual resistivity decreases with an increasing RRR values, consistent with the expectation that RRR reflects the quality of samples.

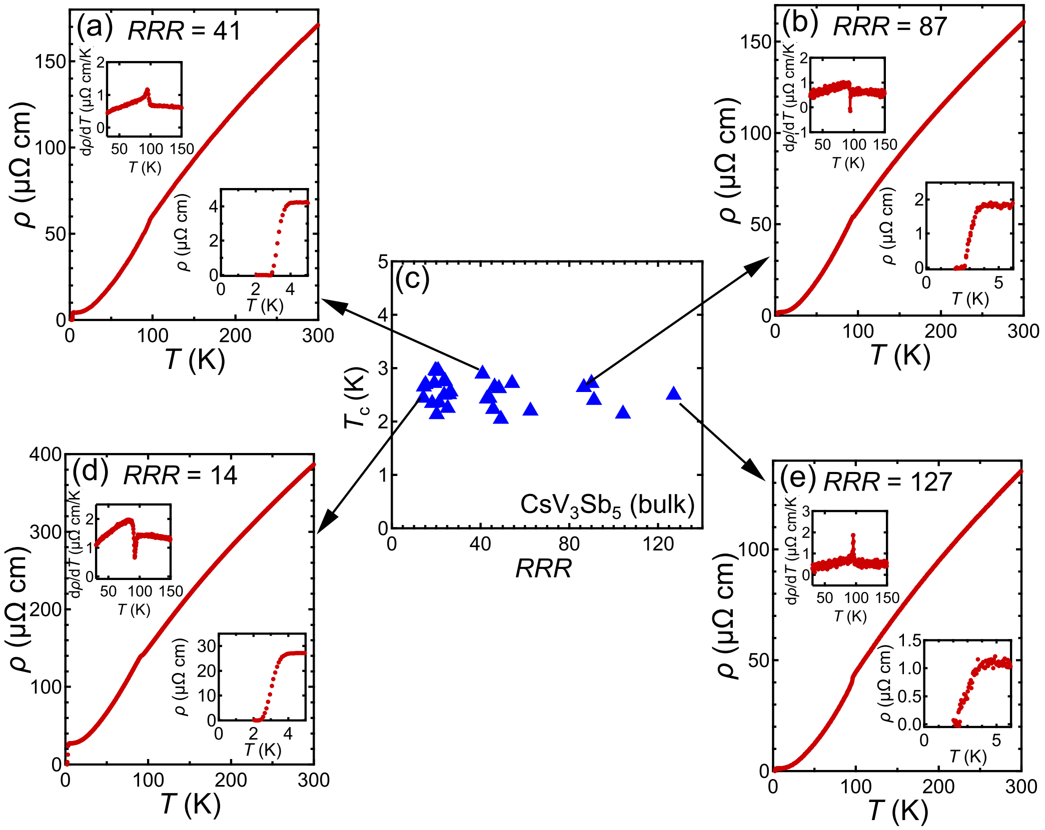

FIG. S1. Temperature dependence of resistivity for selected bulk samples.

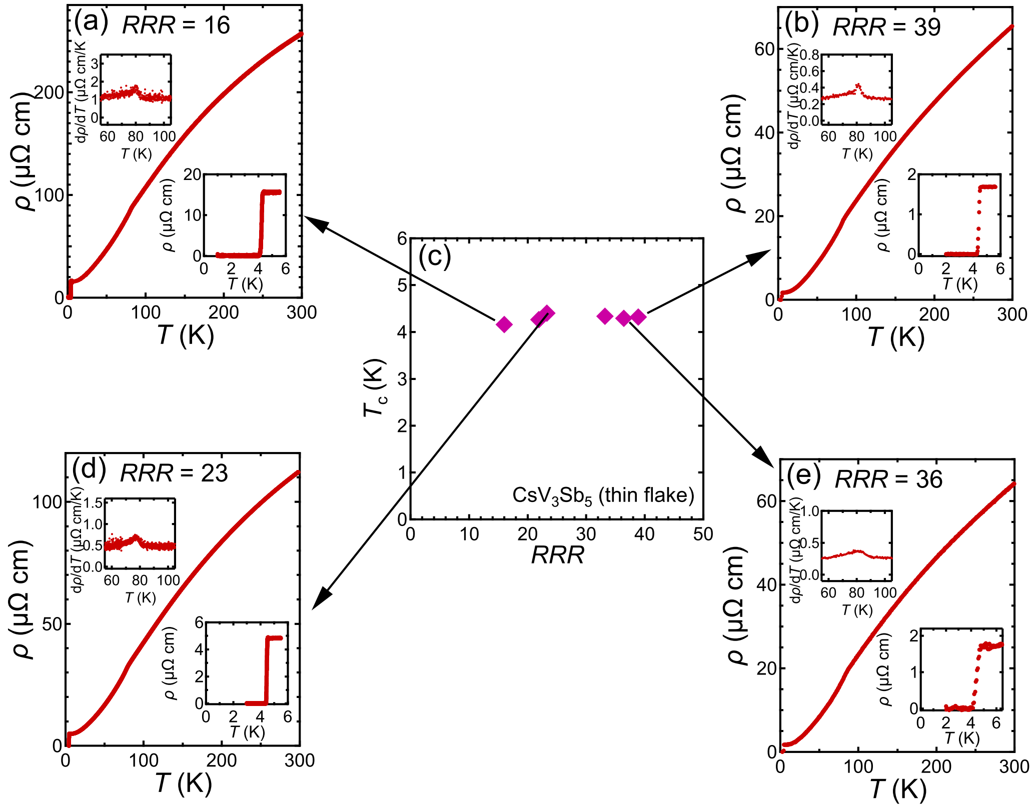

FIG. S2. Temperature dependence of resistivity for selected thin flake samples.

Additionally, we extract T_{CDW} from our resistivity data. Figure S3 shows T_{CDW} vs. RRR in the bulk samples. The RRR dependence of T_{CDW} is weak.

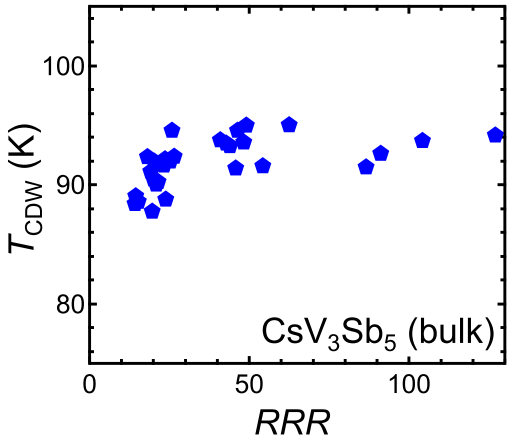

FIG. S3. RRR dependence of T_{CDW}.

Supporting Information S2:

Analysis of the dip feature in dV/dI at 39.9 kbar

At 39.9 kbar, additional features appear in dV/dI, which might indicate inhomogeneity or the presence of the second gap. We notice a sharp dip in dV/dI beyond I_c (see the short arrow in Fig. S4 (a)), which is equivalent to a flat region in the V-I curve. We track the temperature dependence of the dip feature, and find that the feature can be described by a single s-wave gap.

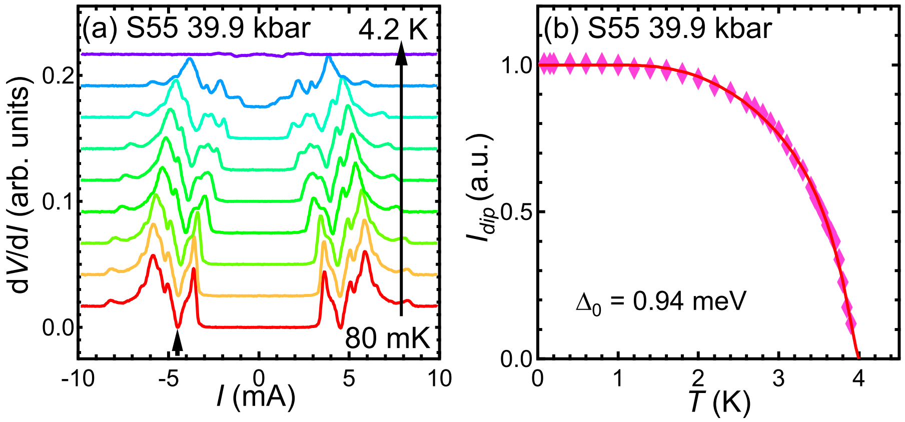

FIG. S4. (a) The calculated first derivative of $V(I)$, dV/dI, of S55 at 39.9 kbar. The short black arrows indicate the position of the dip on the negative current side. We define this feature as I_{dip}. (b) Temperature dependence of the normalized I_{dip}. The solid curve is the single s-wave gap fit, which gives the superconducting gap value of 0.94 meV (2.73 $k_B T_c$).

Supporting Information S3:

Comparison of the superconducting gap with previous studies

In Table S1, we compare the superconducting gap revealed by our self-field critical current with various measurements, including scanning tunneling spectroscopy (STS) [S1–S3], Muon spin rotation (μSR) [S4–S6], specific heat [S7], tunnel diode oscillator (TDO) [S7, S8], soft point-contact spectroscopy (SPCS) and mechanical point-contact spectroscopy (MPCS) [S9, S10]. The entries in red are calculated based on the knowledge of T_c and either Δ or $2\Delta/k_BT_c$. The notation "Δ_i" denotes the value of the ith gap if the corresponding study reported the existence of multiple superconducting gaps. The gap values revealed by our studies are consistent with the soft point-contact spectroscopy measurements, but larger than other studies. However, the T_c in our thin flakes is also relatively high, which may result from a possible orbital selective hole doping mechanism as mentioned in the main text. Hence, the comparison of the gap-to-T_c values among different studies should be more reasonable. As shown in the table, the gap-to-T_c values revealed by our study are consistent with most studies. Importantly, all these techniques (including ours) point to the strong-coupling nature of the superconductivity in CsV_3Sb_5.

Measurement	T_c (K)	superconducting gap (meV)	$2\Delta/k_BT_c$
**This work**	4.0 (M1)<br>4.4 (M4)	Δ=0.95 (M1)<br>Δ=1.08 (M4)	5.4 (M1)<br>5.7 (M4)
STS [S1]	2.6	Δ_1=0.48, Δ_2=0.36, Δ_3=0.38 (Sb surface)<br>Δ_1=0.57, Δ_2=0.30, Δ_3=0.45 (half-Cs surface)	4.3, 3.2, 3.4 (Sb surface)<br>5.1, 2.7, 4.0 (half-Cs surface)
STS [S2]	3	Δ=0.4	3.1
STS [S3]	2.8	Δ=0.52	5.2
μSR [S4]	2.7	Δ_1=0.57, Δ_2=0.23	4.9, 2.0
μSR [S5]	2.5	Δ_1=0.3, Δ_2=0.4	2.8, 3.7
μSR [S6]	N. A.	N. A.	1.1, 5.4
Specific heat [S7]	2.7	Δ_1=0.38, Δ_2=0.15	3.24, 1.26
TDO [S7]	2.7	Δ_1=0.33, Δ_2=0.13	2.82, 1.16
TDO [S8]	2.8	Δ_1=0.5, Δ_2=0.3	4.1, 2.5
SPCS [S9]	3.4	Δ_1=1.05, Δ_2=0.45	7.2, 3.1
SPCS [S10]	4.2	Δ_1=1.27, Δ_2=0.4	7.4, 2.3
MPCS [S10]	5.0	Δ=0.4	2.2

TABLE S1. The superconducting gap in CsV_3Sb_5 revealed by various studies.

REFERENCES

[S1] H.-S. Xu, Y.-J. Yan, R. Yin, W. Xia, S. Fang, Z. Chen, Y. Li, W. Yang, Y. Guo, and D.-L. Feng, Multiband superconductivity with sign-preserving order parameter in kagome superconductor CsV_3Sb_5, Phys. Rev. Lett. **127**, 187004 (2021).

[S2] Z. Liang, X. Hou, F. Zhang, W. Ma, P. Wu, Z. Zhang, F. Yu, J.-J. Ying, K. Jiang, L. Shan, Z. Wang, and X.-H. Chen,

Three-dimensional charge density wave and surface-dependent vortex-core states in a kagome superconductor CsV_3Sb_5, Phys. Rev. X **11**, 031026 (2021).

[S3] H. Chen, H. Yang, B. Hu, Z. Zhao, J. Yuan, Y. Xing, G. Qian, Z. Huang, G. Li, Y. Ye, *et al.*, Roton pair density wave in a strong-coupling kagome superconductor, Nature **599**, 222 (2021).

[S4] R. Gupta, D. Das, C. H. Mielke III, Z. Guguchia, T. Shiroka, C. Baines, M. Bartkowiak, H. Luetkens, R. Khasanov, Q. Yin, *et al.*, Microscopic evidence for anisotropic multigap superconductivity in the CsV_3Sb_5 kagome superconductor, npj Quant. Mater. **7**, 49 (2022).

[S5] R. Gupta, D. Das, C. Mielke, E. T. Ritz, F. Hotz, Q. Yin, Z. Tu, C. Gong, H. Lei, T. Birol, R. M. Fernandes, Z. Guguchia, H. Luetkens, and R. Khasanov, Two types of charge order with distinct interplay with superconductivity in the kagome material CsV_3Sb_5, Commun. Phys. **5**, 232 (2022).

[S6] Z. Shan, P. K. Biswas, S. K. Ghosh, T. Tula, A. D. Hillier, D. Adroja, S. Cottrell, G.-H. Cao, Y. Liu, X. Xu, Y. Song, H. Yuan, and M. Smidman, Muon spin relaxation study of the layered kagome superconductor CsV_3Sb_5, Phys. Rev. Research **4**, 033145 (2022).

[S7] W. Duan, Z. Nie, S. Luo, F. Yu, B. R. Ortiz, L. Yin, H. Su, F. Du, A. Wang, Y. Chen, *et al.*, Nodeless superconductivity in the kagome metal CsV_3Sb_5, Sci. China: Phys. Mech. Astron. **64**, 107462 (2021).

[S8] M. Roppongi, K. Ishihara, Y. Tanaka, K. Ogawa, K. Okada, S. Liu, K. Mukasa, Y. Mizukami, Y. Uwatoko, R. Grasset, *et al.*, Bulk evidence of anisotropic s-wave pairing with no sign change in the kagome superconductor CsV_3Sb_5, 2022, 2206.02580, arXiv, https://arxiv.org/abs/2206.02580 (Accessed September 18th, 2022).

[S9] M. He, H. Zi, H. Zhan, Y. Zhao, C. Ren, X. Hou, L. Shan, Q. Wang, Q. Yin, Z. Tu, *et al.*, Strong-coupling superconductivity in the kagome metal CsV_3Sb_5 revealed by soft point-contact spectroscopy, Phys. Rev. B **106**, 104510 (2022).

[S10] L. Yin, D. Zhang, C. Chen, G. Ye, F. Yu, B. R. Ortiz, S. Luo, W. Duan, H. Su, J. Ying, *et al.*, Strain-sensitive superconductivity in the kagome metals KV_3Sb_5 and CsV_3Sb_5 probed by point-contact spectroscopy, Phys. Rev. B **104**, 174507 (2021).
